# Racial/ethnic differences in the prevalence and incidence of metabolic syndrome in high-income countries: a protocol for a systematic review

**DOI:** 10.1186/s13643-020-01400-y

**Published:** 2020-06-08

**Authors:** Nicholas Kofi Adjei, Florence Samkange-Zeeb, Mihiretu Kebede, Maham Saleem, Thomas L. Heise, Hajo Zeeb

**Affiliations:** 1 Department of Prevention and Evaluation, Leibniz Institute for Prevention Research and Epidemiology - BIPS, Achterstrasse 30, D-28359 Bremen, Germany; 2 Health Sciences Bremen, University of Bremen, Bremen, Germany

**Keywords:** Metabolic syndrome (MetS), Race, Ethnicity, Migrants, High-income countries

## Abstract

**Background:**

Metabolic syndrome is a constellation of various cardiovascular and type 2 diabetes risk factors, such as abdominal obesity, atherogenic dyslipidemia, high blood pressure, and high blood glucose, but its prevalence varies widely by geographical region, sex, and race/ethnicity. The objective of this study is to examine the prevalence and incidence of metabolic syndrome among adults of different racial/ethnic origins in high-income countries.

**Methods:**

We designed and registered a study protocol for a systematic review of descriptive epidemiological data. Observational studies (e.g., cross sectional and cohort studies) reporting morbidity data of metabolic syndrome and conducted in a wide range of adult people (e.g., different racial/ethnic origins, including migrants) will be included. The primary outcome will be the prevalence and incidence of metabolic syndrome. Secondary outcomes will be the prevalence and incidence of individual components of metabolic syndrome (e.g., abdominal obesity, dyslipidemia, high blood pressure, and high blood glucose). Literature searches will be conducted in several electronic databases (from inception onwards), including MEDLINE, Web of Science Core Collection (Science Citation Index and Social Science Citation Index), CINAHL, and Cochrane Library. Two investigators will independently screen all reference titles, abstracts, and full-text articles. The methodological quality (or potential bias) of selected studies will be appraised using an appropriate tool. Our results will be described narratively. Random-effects meta-analysis will be conducted, if feasible and appropriate. Additional analyses will be conducted to explore the potential sources of heterogeneity.

**Conclusion:**

This systematic review will identify, evaluate, and integrate prevalence and incidence data of metabolic syndrome, with focus on racial/ethnic differences in high-income countries. We anticipate our findings may guide policy formulation and identify knowledge gaps in the literature that future research should address.

**Systematic review registration:**

PROSPERO, CRD42020157189

## Background

Metabolic syndrome (MetS) is a clustering of metabolic and physiological abnormalities [1], including hyperglycemia, obesity, atherogenic dyslipidemia, and hypertension [1, 2]. As MetS is not a single pathogenic entity, but rather an array of risk factors for cardiovascular and metabolic diseases [3, 4], various institutions have defined it differently [5–7]. Among these institutions are the World Health Organization (WHO) [8], International Diabetes Federation (IDF) [6], National Cholesterol Education Program Adult Treatment Panel III (NCEP: ATPIII) [9], and the American Heart Association/National Heart, Lung and Blood Institute (AHA/NLHBI) [5]. There are but some minor variations in the definitions of MetS proposed by the named institutions [6]. At present, the two most widely used definitions are those put forward by the NCEP: ATPIII and the IDF [10].

The worldwide prevalence of MetS among adults is estimated to be 20-25% [11], but there are country and regional variations [12] depending on the definitions used [10]. The National Health and Nutrition Examination Survey (NHANES) in the USA estimated the prevalence of MetS to be 34.5%, based on the NCEP: ATPIII criteria [13]. Australia has a prevalence of 22.1% using the NCEP: ATPIII definition and 30.7% using the IDF definition [14]. In Europe, the overall prevalence has been reported to be 24.3% according to the NCEP: ATPIII definition [15].

There is however evidence that the prevalence and incidence of MetS differ between specific populations (i.e., by sex, race, and ethnicity) in high-income countries [13, 16]. Previous studies in Europe suggest a higher prevalence of MetS among migrants/ethnic minorities than host populations [17]. In the USA, some racial/ethnic differences have also been noted [18, 19]. Whereas some studies reported higher prevalence of MetS in non-Hispanic Whites compared to Black populations [20, 21], other findings suggest that Hispanics are at an increased risk of developing MetS than non-Hispanic Whites [22, 23]. The patterning of these inequalities are complex, and several studies implicate genetic variation, environmental and socio-economic factors as contributing factors to the possible causes of racial/ethnic differences in cardiovascular and metabolic diseases [24, 25]. A better understanding of MetS prevalence and incidence among diverse racial/ethnic groups is crucial for the inequalities in metabolic diseases to be addressed.

The objective of this study will be to systematically evaluate observational epidemiological studies that present prevalence and incidence data of metabolic syndrome among adults of different racial/ethnic groups. The research question that will be addressed is—to what extent do incidence and prevalence of metabolic syndrome differ among racial/ethnic groups of adults in high-income countries?

## Methods

The present protocol has been registered within the PROSPERO database (Registration ID: CRD42020157189). This study protocol is being reported in accordance with the reporting guidance provided in the Preferred Reporting Items for Systematic Reviews and Meta-Analyses Protocols (PRISMA-P) statement [26] (see PRISMA-P checklist in Additional file 1). The proposed systematic review will be reported in accordance with the reporting guidance provided in the Preferred Reporting Items for Systematic Reviews and Meta-analyses (PRISMA) statement [27] and the Meta-analysis Of Observational Studies in Epidemiology (MOOSE) reporting guideline [28].

### Inclusion and exclusion criteria

Studies will be selected according to the following criteria: study design (and context), participants, exposures, and condition or outcome(s) of interest.

### Study design and context

Eligible studies will be quantitative, observational studies (cohort, cross-sectional or health surveys) reporting prevalence and/or incidence data using validated and non-validated tools and conducted in a wide range of people in the general, non-institutionalized population (e.g., including data from administrative databases and registries) from high-income countries. Cross-sectional studies will be the most appropriate study design to determine the prevalence of metabolic syndrome, and cohort studies will be the most appropriate study design to determine the incidence of metabolic syndrome. Cross-sectional health surveys are typically used to estimate the point prevalence of common conditions of long duration. For cohort studies, both the first phase (cross sectional) data and follow-up phase will be considered. We will exclude studies in hospital/inpatient clinical settings because they are likely to be highly selected (selection bias), resulting in inaccurate estimations of the “true prevalence and incidence” of the metabolic syndrome across different groups in the general population. In addition, we will exclude reviews, case reports, case series, qualitative studies, and opinion articles. However, we will use review articles to identify any potential studies that might have been missed from our search.

### Participant (population)

We will include studies involving adult populations (≥ 18 years old), regardless of sex and race/ethnicity in high-income countries, as classified by the Organization for Economic Co-operation and Development (OECD) [29].

### Exposures and comparators

The main exposure in this study will be race/ethnicity and immigration status. We will define a priori the following racial/ethnic groups [e.g., White/European, Hispanic/Latin American, Black/African (e.g., Sub-Saharan African and Caribbean), Asian, and Arab]. Studies in a homogeneous population with diverse tribal groups will be excluded. Studies comparing migrants and host populations will be included. Since there is heterogeneity in how migrants are labeled, we will follow the general conventions used globally [30]. In studies reporting comparisons between racial/ethnic groups, the comparator will be the “majority” groups. For example, in the USA, the majority ethnic group would be “White,” while the minority ethnic group would be “Black” “Hispanic” “Asian,” and “other” ethnic backgrounds [31]. In Europe, the minority ethnic group will generally also include people with a migration background [32].

### Outcomes

The primary outcome will be the prevalence and incidence of metabolic syndrome. We will use author-reported definitions (according to accepted diagnostic criteria, but also self-reported). According to the harmonized definition [33], diagnosis of metabolic syndrome requires the fulfillment of at least 3 of the following 5 criteria: waist circumference ≥ 102 cm in men and ≥ 88 cm in women; fasting blood glucose level ≥ 100 mg/dL or treatment with antidiabetic drugs; systolic or diastolic blood pressure ≥ 130 mmHg or ≥ 85 mmHg, respectively, or treatment with antihypertensive medication; triglyceride level ≥ 150 mg/dL; and serum high-density lipoprotein cholesterol level < 40 mg/dL in men or < 50 mg/dL in women.

Secondary outcomes will be the prevalence and incidence of individual components of metabolic syndrome, such as abdominal obesity, dyslipidemia, high blood pressure, and high blood glucose.

### Information sources and search strategy

The primary source of the literature will be a structured search of major electronic databases (from inception onwards): MEDLINE (Ovid), Web of Science Core Collection (the Social Science Citation Index [SSCI], the Science Citation Index [SCI]), the Cumulative Index to Nursing and Allied Health Literature (CINAHL), and Cochrane Library. We will also perform hand-searches of the reference lists of included studies, relevant reviews, clinical practice guidelines or other relevant documents. Further, content experts and authors who are prolific in the field will be contacted. The literature searches will be designed and conducted by the review team with the help of a health information specialist. A draft search strategy for MEDLINE is provided in Additional file 2. No limitations will be imposed on language, publication status, and study conduct period. The search results will be uploaded to an online reference management tool (EndNote X9.2 reference manager).

### Screening and selection of studies

All references identified from the literature search will be imported into Covidence [34], a web-based software that aids the management of systematic reviews. This software will further be used for the title/abstract screening. First, titles and abstracts of references returned from initial searches will be screened independently by two reviewers (NKA and FSZ), based on the eligibility criteria outlined above. Second, full texts will be examined in detail and screened for eligibility by the two reviewers. Third, the references of all the included studies will be hand-searched to identify any relevant report missed in the initial searches. Any disagreements between the two reviewers will be resolved by discussion to meet a consensus. A flow chart showing details of studies included and excluded at each stage of the study selection process will be provided.

### Data extraction

Data extraction will be done independently by the two reviewers (NKA and FSZ), in a pre-piloted data extraction form created in MS Excel. Any discrepancies in the extracted data will be resolved by consensus or discussion with a third reviewer (HZ). The following details will be extracted from each study: (i) details of the study (first author’s last name, year of publication, country), (ii) study design (study design, sample size, sampling method, ethnic group, age, and gender of participants), (iii) metabolic syndrome definition criteria, (iv) frequency, incidence, and prevalence of metabolic syndrome and its components for all adults.

### Quality assessment and risk of bias

The risk of bias in the included studies will be assessed by two review authors (NKA and FSZ), using the Effective Public Health Practice Project (EPHPP) assessment tool for quantitative studies [35]. The EPHPP assess different components of study validity: study design, confounders, selection bias, blinding, data collection method, and dropouts. The overall methodological quality will be rated as strong, moderate or weak. A third reviewer (HZ) will be consulted should there be differences in opinion.

### Data synthesis and analysis

The data from each study (i.e., prevalence and incidence) will be used to construct evidence tables of an overall description of the included studies. If the studies are diverse and quantitative syntheses is not feasible, we will consider presenting the reported studies using albatross plots, following the methodological guideline by Harrison and colleagues [36]. Heterogeneity across studies with respect to characteristics of studies such as design, population, and methodological difference will first be qualitatively evaluated. Secondly, statistical heterogeneity will be quantified using I-square and Tau-square statistics. Cochrane chi-square test will be used to evaluate statistical heterogeneity at 10% level of significance. If meta-analysis is feasible and studies are found to be combinable, random-effects meta-analysis of prevalence/incidence data will be conducted for primary and secondary outcomes. We anticipate a sizable statistical heterogeneity across studies; hence, we will estimate the pooled prevalence using the random-effects model. If sufficient studies are identified and data points are available, we will investigate potential sources of heterogeneity, and forest plots will be used to visualize the extent of heterogeneity among studies. We plan to conduct subgroup analysis by gender (male vs. female).

### Meta-biases

If the data permits, publication bias across individual studies will be assessed by visually inspecting the asymmetry track pattern on the funnel plot and by Egger’s test [37]. In addition, the impact of risk of bias of individual studies on the overall effect size will be assessed by conducting meta-regression and subgroup analysis. We will compare pooled effect sizes for subgroup of studies (i.e., studies with high risk of bias vs low risk of bias).

## Discussion

MetS and its components, namely, central obesity, raised blood pressure, hyperglycemia, and dyslipidemia [1, 2, 38] have been identified as risk factors for type 2 diabetes [39, 40], cardiovascular diseases [3], and coronary heart disease-related mortality [41]. To our knowledge, this will be the first review to systematically synthesize and collate the available evidence on the incidence of prevalence of MetS in different racial/ethnic groups, including migrant populations. The results will provide an opportunity for interventions to avert the development of cardiovascular diseases among certain racial/ethnic groups [42], especially ethnic minorities [32]. The findings may further guide policy formulation and also highlight gaps in the literature that need to be considered in future research. The review findings will be made publicly available, thus any amendment made to this protocol will be outlined and reported in the final manuscript and the PROSPERO database.

The proposed study may have some limitations at the study and review level, inter alia, expected heterogeneity in the individual studies, quality of the study designs and definition, and assessment of the primary outcome.

## Supplementary information

**Additional file 1:.** PRIMA-P 2015 checklist

**Additional file 2:.** Medline Search strategy

## Data Availability

Not applicable

## Abbreviations

CINAHL: Cumulative Index to Nursing and Allied Health Literature
EPHPP: Effective public health practice Project
IDF: International Diabetes Federation
MetS: Metabolic syndrome
NCEP: ATPIII: National Cholesterol Education Program Adult Treatment Panel III
NHANES: National Health and Nutrition Examination Survey
OECD: Organization for Economic Co-operation and Development
PRISMA: Preferred Reporting Items for Systematic Reviews and Meta-Analyes
SCI: Science citation index
SSCI: Social science citation index
WHO: World Health Organization
