# Supplementary material for: Racial/ethnic differences in the prevalence and incidence of metabolic syndrome in high-income countries: a protocol for a systematic review
Source: Syst Rev. 2020 Jun 8;9:134. doi: 10.1186/s13643-020-01400-y (PMC7282095; doi:10.1186/s13643-020-01400-y)
Supplement: Supplementary file 2 — Additional file 2:. Medline Search strategy [file 13643_2020_1400_MOESM2_ESM.docx]

*Table S1.* Medline Search Strategy

| Line | Search | |
| --- | --- | --- |
| 1 | | ((race* or racial or ethnic* or ethnicit*) adj5 minorit*).ti,ab. |
| 2 | | ((raci* or race or ethnic* or minorit* or immigra* or emigra* or migrant* or migration) adj5 (group* or communit* or population*)).ti,ab. |
| 3 | | exp minority groups/ |
| 4 | | exp minority health/ |
| 5 | | exp ethnic groups/ |
| 6 | | exp "emigrants and immigrants"/ |
| 7 | | or/1-6 |
| 8 | | METS.ti,ab. |
| 9 | | metabolic syndrome.ti,ab. |
| 10 | | exp metabolic syndrome/ |
| 11 | | or/8-10 |
| 12 | | 7 and 11 |
| 13 | | ((race* or racial or ethnic* or raci* or immigra* or emigra* or migrant* or migration or minorit*) adj15 (METS or metabolic syndrome)).ti,ab. |
| 14 | | 12 or 13 |
